# Dancing in a culture of disordered eating: A feminist poststructural analysis of body and body image among young girls in the world of dance

**DOI:** 10.1371/journal.pone.0247651

**Published:** 2022-01-12

**Authors:** Nicole Doria, Matthew Numer

**Affiliations:** School of Health and Human Performance, Stairs House, Dalhousie University, Halifax, Nova Scotia, Canada; Sapienza - University of Roma, Italy, ITALY

## Abstract

Eating disorders among adolescent girls are a public health concern. Adolescent girls that participate in aesthetic sport, such as dance, are of particular concern as they experience the highest rates of clinical eating disorders. The purpose of this study is to explore the experiences of young girls in the world of competitive dance and examine how these experiences shape their relationship with the body; feminist poststructural discourse analysis was employed to critically explore this relationship. Interviews were conducted across Canada with twelve young girls in competitive dance (14–18 years of age) to better understand how the dominant discourses in the world of competitive dance constitute the beliefs, values and practices about body and body image. Environment, parents, coaches, and peers emerged as the largest influencers in shaping the young dancers’ relationship with their body. These influencers were found to generate and perpetuate body image discourses that reinforce the ideal dancer’s body and negative body image.

## Introduction

Eating disorders are a public health issue in North America, where approximately 10% of young girls struggle with an eating disorder [1]. Young girls who participate in aesthetic sports, such as dance, are at particularly high risk for developing clinical eating disorders because of the additional and extreme pressures to be thin. In the world of dance, the pressures to be thin are enforced through the image of an ideal dancer’s body, encouragement and support of a low body weight, the use of mirrors as a teaching tool, and performing on stage to be observed and evaluated [2–5]. When compared to non-dancers, female dancers are known to suffer from greater body image dissatisfaction, body image distortion, neurotic perfectionism, and disordered eating [3–6]. Many female dancers also suffer from the associated health issues of eating disorders, such as amenorrhea, irreversible bone loss, digestive problems, anxiety, depression, and decreased self-esteem [4, 5]. There is a relatively high incidence of eating disorders and eating disorder behaviours (EDBs) among female dancers, and there is potential for female dancers to battle EDBs and the associated health consequences across their lifetime [7, 8].

### The work of feminist writers

Feminist writers have long emphasized the critical role of Westernized sociocultural values in the development of EDBs. Orbach [9] presents some of the earliest work in this area, with her book *Fat is a Feminist Issue*. Orbach [9] asserts that women’s bodies come in all shapes and sizes, but this variety is systematically ignored in Western culture. This makes women feel alienated from their bodies that are “oversized” when compared to the dominating images of slimness. This culture, Orbach [9] argues, is obsessed with thinness, and defines femininity by body image. Orbach [10] further believes that compulsive eating, bulimia, and anorexia are the psychological response to these cultural beliefs around body and body image.

In the 1990s, there was a surge of new feminist writings on the body [11–14]. Bordo [12] is arguably the most influential author of this time with her work *Unbearable Weight*: *Feminism*, *Western Culture*, *and the Body*. Maintaining a focus on cultural influences, Bordo [12] argues that the women’s body is profoundly gendered, and the gendered nature of mind/body dualism is embodied in contemporary culture. Bordo [12] denies that the body is purely physiological and recognizes that historically the bodies of women have been constantly in the grip of cultural practices. Bordo [12] also addresses the fear and disgust of fat that pervades contemporary culture in all forms (advertisements, movies, music, literature), which contributes to the cultural production of eating disorders; stating that “eating disorders, far from being “bizarre” and anomalous, are utterly continuous with a dominant element of the experience of being female in this culture” (p. 57). Bordo [12] believes most women, regardless of age, race, or ethnicity, suffer from disordered eating. According to Bordo [12], women fall on a continuum that ranges from dieting to clinical eating disorders.

Hesse-Biber is another contemporary feminist writer who focuses on the impact of sociocultural factors regarding women and the body. In her most recent work, *The Cult of Thinness*, Hesse-Biber [15] is the first to present the cult analogy. She compares the intense obsession of being thin with a religious cult that is strongly devoted to their beliefs. Hesse-Biber [15] describes the primary membership for the cult of thinness as being a woman and the object of worship for the members as the perfect body: “They may not answer to a single leader, but bow instead to powerful economic, social and cultural forces that define and value females in terms of their physical attributes” (p. 15). The women of this cult engage in ritualisticbehaviours of dieting, exercising, counting calories, and obsessive weighing of their bodies. Members swear total allegiance to the scale and its verdict determines self-worth, emotional state, and future behaviours. These common rituals, along with the belief that being thin is sacred and the key to happiness, bring them together and keep them faithful to the cult. Hesse-Biber [15] recognizes that most young women are believers, and the severity of their disordered eating behaviours is dependent on where they fall on the continuum of disordered eating.

### Adolescent girls and eating disorders

Especially during adolescence, girls are under great pressure to conform to gendered beliefs and cultural expectations [16]. Western society today reinforces that power for girls is about being sexually attractive and encourages young girls to become obsessed with their weight and appearance, which makes young girls particularly vulnerable to the development of EDBs [16–18]. Once a disorder that was thought to only affect the white middle class, is now known to affect young girls regardless of ethnicity, race, or class [15, 19, 20]. The literature that takes a feminist approach to weight preoccupation and eating disorders in young girls is concerned with cultural factors that create an environment where physical appearance is deeply valued [21, 22]. This literature tends to largely focus on the thin ideal and objectification.

#### The thin ideal

Body dissatisfaction, which is associated with a heightened risk for the development of EDBs, has increased in recent decades among adolescent girls [23, 24]. This has largely been attributed to the internalization of the thin body ideal, which is a socially defined ideal of attractiveness that is often unrealistic and fostered by the pressure to be thin [25]. The oppression of women and young girls to obtain the thin ideal is a post 1960s phenomena. The blame is often placed on the influence of the fashion industry because the 1960s is when slenderness became the pinnacle of fashion [26].

The women’s body has not altered for thousands of years. What has altered is the ideal, and never has this ideal been a body so “close to the bone” [26]. Bordo [12] notes that in Western culture, "fat is the devil, and we are continually beating him—’eliminating’ our stomachs, ’busting’ our thighs, ’taming’ our tummies—pummeling and purging our bodies, attempting to make them into something other than flesh" (p. 107). This contemporary “tyranny of slenderness” affects far more women than it does men, which is evidenced by women being more obsessed and less satisfied with their bodies. Women are also permitted less freedom regarding their weight, particularly when they are young [21].

Adolescent girls are the most likely to buy into this ideal and engage in behaviours to produce it [17]. Evidence suggests that body image and dissatisfaction is a principal determinant of adolescent food choices [27]. Given that many adolescent girls strive to achieve this ideal, they engage in weight loss behaviours to achieve it [28]. Young girls feel a great deal of conflict when they know that the desired image is thin and good and the calories in the food are bad [29]. The division of food into “good” and “bad” categories is a result of food watching. Hesse-Biber [15] found “good foods are nutritionally sound and or/low in calories. Bad foods are the ones that tempt you to overeat and gain weight. They include sweets, refined carbohydrates, and anything with the “F-word”, fat” (p. 146). Disciplined eating and restricting food intake are often maintained through calorie counting, meal skipping and dieting for the purpose of controlling weight [15]. These behaviours are shown to be common in young girls [27, 30]. It has also been found that adolescent girls wrongly perceive dieting to mean the same thing as healthy eating [31]. This is further supported in a study by Lattimore and Halford [32], which compared female adolescents who dieted with female adolescents who were non-dieters. It was found that female adolescents who dieted made generally healthier food choices such as an increase in fruit and vegetable consumption.

Young girls believe that their bodies and their weight are easy to access and manipulate. As a result of this belief, EDBs can be used as a tool for accomplishing the ideal body [15]. Perhaps EDBs are the only tool that young girls have to satisfy the societal ideals dictated by culture [15, 17]. Young girls learn that their body is as an instrument with the sole purpose of being thin and attractive for the consumption of others, and mostly for the consumption of men [33]. Hesse-Biber [15] stresses that this concept of body and EDBs as a “tool” is important for understanding the prevalence and increase of EDBs in young girls.

The work of Ortner [34] and Hesse-Biber [15] discuss the role of patriarchy in this male/female dichotomy that causes women to become obsessed with the thin ideal. Women have been historically dependent on male approval for their survival, and both judged and rewarded for their appearance. It is therefore not surprising that young women want to have a body that appeals to men [35]. For young girls today, consumption of media and social media are great enforcers of the thin ideal and subsequently produce a drive for thinness that influence food and body choices [15, 17, 21].

#### Media

Westernized sociocultural values that associate thinness with beauty, popularity, happiness, and success are transmitted through almost all media messages [36]. Media messages that encourage thinness have been found to have more influence on the development of negative body image in girls than any other influencer (family, friends, and peer pressure) [37, 38] and create an additional risk factor for the development of EDBs in adolescent girls [39, 40]. Research suggests that girls as young as seven internalize media messaging regarding their bodies [41]. Young girls embody and enforce this ideal and constrain themselves within the confines of this image. They are vulnerable and powerless to the influence of advertising and unable to resist the impossible, yet desirable, images presented to them [42].

In recent years, adolescents have been affected not only by media in magazines and television but also by messaging from social media. It has been found that the more time adolescent girls spend on Facebook, the higher their risk for development of EDBs [43, 44]. In 2007, approximately 60% of adolescents aged 12–17 were on social networks. By 2009, this number grew to 75% of adolescents [45]. Today, 96% of Canadian youth aged 16–24 use social media daily with a quarter checking their smartphones constantly [46]. The increasing number of adolescents on social media is consequently increasing the vulnerability of adolescent girls to adopt negative body image and disordered eating attitudes and behaviours [47, 48]. A systematic review conducted in 2016 found that use of social media was associated with body image concerns and EDBs [49].

#### Objectification

Frederickson and Roberts’ [33] Objectification Theory argues that in Western society sexual objectification of girls and women is not just in the media. Constant sexual objectification and objectifying gaze socializes girls and women to view themselves as objects to be evaluated based on appearance. This leads to self-objectification, where an observer’s perspective of the physical self is embodied. Self-objectification manifests itself in the form of habitual self-monitoring/self-surveillance. This behavior has been linked to increased body shame, appearance anxiety, and a decreased awareness of internal bodily states such as hunger. Objectification Theory suggests that the four experiential consequences (shame, anxiety, peak motivational states, and awareness of internal bodily states) combine to put young girls at an increased risk for the development of EDBs [33].

Adolescence is a critical period for the development of self-objectification because it is the onset of puberty. For girls, puberty is the time when the body begins to develop and becomes increasingly looked at and commented on. The body is also more likely to be evaluated by others during or after puberty [33]. Girls become the object of the male gaze when they develop more mature bodies, and it becomes evident to them that they are now an icon of sexuality for male consumption [17]. Furthermore, young girls tend to become more concerned with their image as the pressure to achieve societal norms regarding ideal weight and shape is heightened [50]. Adolescence is when girls make the transition from a relatively androgynous body to a woman’s body, which is a body often devalued in the society and culture they inhabit [33]. Adolescence with anorexia often express a fear of growing into a mature, sexually developed body, because it is associated with disgusting womanly fat [21]. Overall, young women are likely to experience their bodies as the enemy and attempt to dissociate themselves from their bodies during adolescence. This creates a disjointed and contradictory subjectivity [17].

#### Parents and peers

It has been suggested that EDBs are influenced by the quality of relationships with parents and peers [51, 52]. Family functioning specifically has been examined as a risk factor for adolescent girls’ development of EDBs [53–56]. It has been found that families have a strong influence on the feelings associated with one’s body, one’s choices about eating, and the first understandings of body image [56, 57]. Family practices, such as frequency of family meals, parental weight-teasing, perceived importance of thinness to parents, and maternal dieting behaviours have all been found to influence the development of EDBs among adolescent girls [58, 59].

Homes with high-levels of parent-lend conversations about weight and weight-based teasing have been shown to increase frequency of EDB development in adolescent girls [59]. Perceived family functioning may also be associated with the development of EDBs and the severity of symptoms [53]. Cernigilia et al. [53] found that among adolescent girls diagnosed with EDBs, low levels of perceived family functioning increased the reported severity of their disordered eating practices [53].

Contrarily, frequent family meals have been shown to reduce the likelihood of adolescent girls initiating purging behaviours, binge eating, and frequent dieting [58]. Adolescent girls who reported high levels of family support have also been shown to have higher levels of body satisfaction and lower levels of EDBs compared to girls with lower levels of perceived family support [56]. Parental support during adolescents may therefore serve as a protective factor against the development of EDBs [51]. Further, girls with higher attachment levels to their mothers at the ages of 10–16 significantly decreased the likelihood of developing an EDB in later adolescents and perceived attachment to fathers at ages 10–11 predicted less disordered eating behaviours at ages 12–13 [51]. These findings reveal that strong relationships with both parents may serve as a potential defence against the development of EDBs [51].

It is well known that peers also influence social norms and create social standards for appearance and behaviour [60]. The perceived importance of thinness among adolescent’s social relationships has been shown to predict levels of body dissatisfaction and EDBs [60]. Adolescent girls often view being thin as a social advantage based on the perception that their peers hold being thin in high regard [60, 61]; believing that being thin will improve their likability by their peers is one of the highest risk factors for EDB development among adolescent girls [52, 60–62]. Negative body talk among peers, such as teasing and negative comments about one’s weight is another powerful negative reinforcement that has been linked to a wide variety of negative health outcomes, including EDBs [63]. Adolescents who reported higher levels of EDBs also reported higher levels of peer-weight teasing and weight-talk among their friends [61]. When adolescents feel alienated from their peers, there is a higher risk of developing EDBs, as well as an increased severity of symptoms [55]. On the contrary, perceived social support from peers has been found to contribute to having a positive body image and is also a protective factor against EDBs among adolescent girls [60].

### Eating disorders and young girls in dance

For decades, a widely studied group of athletes has been dancers. Although the risk association between the world of dance and EDBs has been well documented in the literature, only recently has research began to examine why this risk association exists; the key elements in the world of dance that make dancers a high-risk group for EDBs remains uncertain [64]. Further, few studies have focused on what creates such a high risk for development and maintenance of EDBs in the world of dance. Existing literature has investigated objectification, competitiveness, and pressure from coaches and peers as potential contributors.

### Objectification

Slater and Tiggemann [65] were the first researchers to examine the components of Objectification Theory in a sample of adolescent girls who were dancers. Contrary to what Slater and Tiggemann [65] predicted, the study found that there was no difference between the adolescent dancers and non-dancers on self-objectification or any of the proposed consequences: body shame, appearance anxiety and disordered eating. These results also contradicted the findings of Tiggemann and Slater [66], which found that adult former recreational dancers scored higher on self-objectification and all its proposed consequences when compared to former non-dancers. Due to this contradiction in findings, Slater and Tiggemann [65] concluded that the lack of difference between the groups was likely not a result of recreational level training. It was concluded that the lack of differences was more likely because all adolescent girls experience exceptionally high levels of objectification and its associated consequences. A second explanation was that the adolescent dancers were significantly thinner than the non-dancer participants and, therefore, closer to societal ideals. As a result of being closer to societally prescribed ideals, adolescent dancers may be less likely to suffer from self-objectification when compared to adolescent girls who are non-dancers.

### Competitiveness

The findings of Slater and Tiggemann [65], which concluded that the lack of difference between dancers and non-dancers was not likely the result of recreational dance training, is contrary to other research findings. The literature generally provides evidence that competition at the elite level places individuals at greater risk for developing EDBs [67]. Regarding competitiveness and female dancers, a study conducted by Thomas, Keel and Heatherton [68] found that in a sample of elite, competitive, and non-competitive ballet dancers, elite dancers demonstrated significantly greater EDBs than dancers in other groups. Similarly, De Bruin, Bakker, and Oudejans [69] looked at 35 adolescent female dancers who spent 16.7 hours a week at dance (and did not participate in any other competitive sport activities to rule out possible influence of other sport environments). A link was found between ego involvement (the emphasis on outperforming others), disordered eating, and competitiveness.

Further, in competitive dance adjudication is an important component, whereas judges are a nonfactor in recreational sport. When the criteria for adjudication stresses thinness, like in dance, young dancers are more prone to engage in disordered eating behaviours [70]. Van Durme, Goossens, and Braet [71] found that competition state anxiety had explanatory value for the dieting behaviour of young dancers. These findings are consistent with the findings of Martinsen et al. [72], who found that feelings of anxiety at the start of competitions may put aesthetic athletes at risk for development of EDBs. It has also been found that the relationship between perfectionist tendencies, which are highly correlated with the development and maintenance of EDBs [73], are especially high during times of stress such as competition season [74, 75]. The desire to become as perfect as possible during competition season may translate into development and maintenance of EDBs in young dancers [76].

### Coaches, parents and peers

High expectations and pressure from parents, coaches and peers are found to be another main factor in the development of EDBs [77]. Weight related pressures have specifically been found to be an important contributor to the development of EDBs in aesthetic athletes, including dancers [68, 78]. Studies that researched environmental pressures of ballet dancers found that coaches, parents and peers were great reinforces of EDBs [70, 79–81]. Kleposki [80] found this was because coaches, parents and peers encourage and support the maintenance of a low body weight. Annus and Smith [79] similarly found that what mediates the relationship between dance and EDBs are comments from teachers and peers about the benefits of dieting. Social comparison between peers, skinfold tests/weigh-ins, and observational learning of dieting also contributed to (EB)Bs in dance [79]. Sundgot-Borgen et al. [81] found the most important determinant to be where, how, and when the dancer is told to lose weight.

When researching adolescent female dancers, Francisco et al. [2] found coaches to be the strongest influencers present in the lives of young dancers. Pressures from coaches to be thin produced pressure on dancers 15–17 years of age to reduce their weight. Interestingly, it was the younger dancers (<15) who reported a higher rate of emotional distress because of weight related pressures and comments. Francisco et al. [2] concluded that older dancers (15–17) no longer felt emotional distress regarding weight related pressures. Older dancers accepted and respected the thinness rule without question, and therefore this pressure no longer caused emotional distress.

### Present study

Despite the significant literature on eating disorders and dancers, research on the prevention of EDBs in dancers is limited [82]. Prevention literature is lacking because previous studies have largely focused on studying the issue as an individual psychological problem, tend to use an objectivist lens, and have focused on collecting quantitative data [82, 83]. Most studies have also solely focused on anorexia in ballet dancers, rather than considering dancers who participate in a variety of dance styles or have other EDBs [76].

The purpose of this study was to explore the following research question: ***How does experience in the world of competitive dance shape the relationship that young girls have with their bodies*?** Based on the limitations of current literature, this study employed a qualitative analysis to better understand the values, beliefs, and practices that the world of dance constitutes around body and body image. Further, the project aimed to uncover the dominant and competing discourses related to body and body image in the world of dance. The findings from this research are intended to elucidate how EDBs in dancers are created and/or resisted, with the goal of better informing prevention initiatives targeted at reducing EDBs in young girls who are competitive dancers.

## Theoretical framework and methods

A qualitative approach was used for this research to allow for an in-depth exploration of the experiences of young girls in competitive dance. A qualitative approach allowed for the dancers’ language to be captured, their feelings to be understood, and their voices to be represented [84, 85]. Specifically, a feminist poststructural approach was chosen to provide a critical lens to explore the beliefs, values, and practices of young dancers [84, 86–88]. This methodology also aimed to provide an understanding of the dominant and competing discourses present in the world of dance and discover how these discourses are constituted, perpetuated, and form ways of knowing in relation to body and body image [86, 89, 90].

### Feminist poststructuralism

Feminist poststructuralism was the theoretical framework employed to explore the beliefs, values, and practices that the world of dance constitutes around body and body image [84, 86, 88, 91–95]. Feminist poststructuralism was born out of liberal feminism, radical feminism, and poststructuralism to better account for the women’s experience [88, 96, 97]. This theoretical framework aims to deconstruct social institutions and power relations that disadvantage and oppress women [88]. For the proposed research, feminist poststructuralism provided a critical lens to examine power; challenge the status quo, victim blaming, and oppression; and explore how women’s experiences in the world of dance are socially and culturally constructed [86, 89, 91, 94, 95].

Beliefs, values, and practices are the concepts considered to better understand and capture women’s experiences in the world of dance. Uncovering the beliefs, values and practices regarding a specific experience is common in research that employs a feminist poststructural framework [86, 98, 99]. Applying beliefs, values and practices to research data also aligns with this project’s proposed analytical approach, feminist poststructural discourse analysis (discussed below), as it ensures “that we stop and pay close attention to a person’s experiences from their perspective and not ours” [86, p. 2258]. Questioning participants subjectivity and agency further elucidated the experiences of young girls in competitive dance by uncovering “how the power relations inherent in any discourse, and between discourses and individuals, affect access to certain knowledge, beliefs and practices” [86, p. 2259].

Foucault [92–94] largely influenced this work and informed the understanding of key concepts such as power, body, and discourse. Foucault views power as relational, situational, shifting and always being negotiated by individuals [88, 93]. He views power relations as something that are always present in human society, and as a place where knowledge is produced [88, 100]. Foucault views the body as the place where the systems of discourse and power relations emerge [88]. He believes that without the body there is no power or subjectivity to script the discourses upon because the body is where discourses inscribe themselves [101]. Foucault [102] discusses this in terms of the docile body: “Something that can be made; out of a formless clay, an inapt body [from which] the machine required can be constructed” (p. 135). Foucault [102] further contends that the body is “pliable, capable of being manipulated, shaped, and trained. It obeys, responds and becomes skillful” (p. 135–136). Foucault recognizes that discourses are ways of constituting knowledge and are more than ways of thinking and producing meaning. Discourses create the body, mind, and emotional life of the subjects they seek to shape [88].

### Participants

All participants (N = 12) were female identifying competitive dancers who ranged in age from 14–18 years (see Table 1 for demographics). Given that this research was only looking at current competitive dancers, 14–18 years range falls within the age of current competitive dancers and reflects the age when EDBs most prevalently become known [103]. A current competitive dancer was defined as a dancer who trains in a structured learning environment, such as a dance studio or professional dance company, and participates in regular competitions and/or performances. Furthermore, a competitive dancer by this definition needed to train for a minimum of 12 hours per week, and ballet training had to account for at least 3 hours of this weekly training. Last, dancers had to be competing for five or more years. This criterion was based on previous studies that found dancers who train in a competitive environment, where ballet is part of their dance training, are more likely to experience EDBs [69–71, 104]. Archinard and Scherer [104] for example found females with five or more years of ballet experience displayed a higher level of psychological symptoms associated with EDBs when compared to females with less than five years of dance training. This was found to be a result of increased exposure to the dance environment [104]. Although this research did not target dancers with EDBs, it was important that dancers had spent enough time in the world of dance to speak to the influence that dance culture had on their relationship with body and body image. This experience may, or may not, have influenced the development of EDBs.

**Table 1:** Demographics of participants.

Participant	Age (Years)	Sex	Location of Competitive Studio (Province)	Competitive Training (Years)	Recreational Training (Years)
P1	18	Female	Ontario	7	4
P2	15	Female	New Brunswick	7	6
P3	16	Female	Ontario	10	3
P4	17	Female	Nova Scotia	12	2
P5	18	Female	Alberta	11	2
P6	17	Female	Ontario	5	10
P7	14	Female	Ontario	6	5
P8	18	Female	Ontario	11	1
P9	16	Female	British Columbia	11	0
P10	14	Female	British Columbia	7	0
P11	14	Female	Alberta	7	3
P12	18	Female	Alberta	8	6

Participants were recruited from competitive dance studios across Canada with the use of purposeful and snowball sampling [105]. The recruitment message to participant was distributed to current competitive dancers and competitive dance environments via Facebook. Participants also provided referrals and shared the Facebook message with their peers. If the interested dancer provided variation to the research, based on their competitive dance environment being different than previous participants interviewed, they were selected for an interview. Besides screening for training location to obtain a diverse sample, participants were selected on a first response basis.

### Procedure

Ethical approval for this study was granted by the Dalhousie Health Sciences Research Ethics Board. The Principal Investigator (PI) explained the study procedure in detail to all dancers, and informed consent was obtained from all participants prior to their participation. The informed consent outlined a detailed account of the study at a 9th grade reading level. This ensured that young participants were sufficiently informed before consenting. The consent form and signature page were provided to participants via email or Facebook as a pdf document prior to scheduling their interview. This gave the individual time to review the consent form and decide if they would like to participate. The parents of some of the younger participants also wanted time to review the consent documents. The signed consent form was obtained prior to the interview, and Doria answered any questions the participants had. Doria also confirmed that the participants had no questions or concerns regarding the consent process before beginning the interview. Given that all interviews were conducted over the phone, written consent from participants was received prior to the interview through email exchange and verbal consent was obtained and recorded before the start of the interview. Third party consent/assent was not required from parents because it was determined by the Research Ethics Board that at age 14 people are able to speak to their own experiences with their bodies, and it was important participants felt autonomous in consenting to do so. Some participants, however, chose to review the documents with their parents and/or guardians. All participants voluntarily agreed to be interviewed and no incentive was provided.

One-on-one semi structured interviews, directed by an open-ended interview guide (see S1 Appendix), were conducted with all participants. Probing questions were also used, which allowed for extraction of detailed data and the ability to ask iterative questions [106]. Interview questions were in relation to the world of dance and its influence on shaping the dancer’s relationship with body and body image. With the participants’ permission, all interviews were conducted over the phone using the recording app *TapeACall*. All interviews lasted between 45 and 90 minutes and participants were offered the opportunity to stop their participation at any time during the interview. Interviews were conducted in a non-judgmental and non-hierarchical conversational manner by Doria, who identifies as a woman and former competitive dancer.

### Data analysis

The audio-taped interviews were transcribed verbatim by Doria and analyzed using discourse analysis. Discourse analysis is a methodology of analyzing text for the purpose of interpreting how language, at a given time and place, is used to reflect reality and construct it to be a certain way [90]. The goal of discourse analysis “is not to ensure the methodological conditions for discovery of truth but to understand the conditions under which differing accounts are produced and how meaning is assumed to be produced from them” [107, p. 350]. Discourse analysis is a critical and reflexive approach to analysis that moves beyond the level of common sense and challenges everyday realities [86, 89].

Interviews were transcribed verbatim after each interview and all identifying information was removed at this time. Interview transcripts were analyzed using discourse analysis as outlined by Aston’s [86] guide to using feminist poststructuralism informed by discourse analysis (see Table 2). Discourse analysis identified beliefs, values, practices, social and institutional discourses, relations of power, and moments of subjectivity and agency to uncover the experiences of participants [86, 88, 89, 108, 109].

**Table 2:** A guide to using FPS informed by discourse analysis [86].

1) Identify important issues	Read the transcript and mark quotations you feel represent an important issue. Name the issue as you see it.
2) Applying beliefs, values and practices	Provide the quotation (cut and paste) and write something about the Belief, Value and Practice within the quotation.
3) Social and institutional discourses	Write about the social and institutional discourses you see informing the issue you identified. Sometimes this is clearly described in the quotation but most often you need to expand on the implied ideas. You still need to clearly connect to the evidence (words and meaning provided by participant).
4) Responding to relations of power	As you write about the discourses, you need to connect these ideas to the participant. How do the discourses affect the participant? Does he/she agree or disagree with the beliefs, values and practices? Is it an easy or positive fit? Or are there questions, conflicts, tensions, etc.? These are the “relations of power” that the participant is feeling/experiencing.
5) Subjectivity and agency	You can also add in the participant’s “subjectivity” (how they are positioned as a nurse, man, woman, teacher etc.) as well as their “agency” (how they choose to act in each situation by fitting in or challenging).

Data analysis involved two researchers (ND, MN) individually reading the transcripts, applying the concepts outlined in Table 2, and coming together to establish themes and subthemes; the analysis was completed without software. A variety of discourses emerged from the data, and all perspectives were openly and equitably regarded and valued [108]. Direct quotes are reported in the findings by participant number and age of the corresponding participant (ex. P1, 17).

## Findings

The findings elucidate the threads of discourse in the world of dance that came to dominance through the interviews. The primary focus is on the discourses related to body and body image, and how these discourses shape the experiences of young girls in competitive dance. Many factors are at play in producing young women’s conceptions of body, but the world of dance utilizes, with great precision, a gaze that governs the body like few other experiences in girls’ lives. The results begin with the environmental factors present, followed by the influence of parents, coaches, and peers.

### Environment

The world of dance is a competitive culture inclusive of several environmental elements including mirrors, dance attire, costumes, and the image associated with the ideal dancer’s body. These environmental elements shape the dancer’s relationship with their bodies and contribute to specific discourse surrounding them. In general, the dance environment creates many barriers that make it difficult for dancers to confidently shape a positive relationship with the body. These environmental elements, however, are predominantly negative because of the culture in which they are situated.

#### Competitive culture

The competitive world of dance creates considerable stress for dancers. Participants who compared the competitive dance environment to the recreational dance environment noted: ‘The competitive culture was more about looking good…where recreational dance was more about having fun and just doing something that you loved…it was a lot more carefree and way less stressful’ (P12, 18). Due to the stress and commitment of the competitive dance environment, participants often compared dancing competitively to having a full-time job. Even when participants were away from the dance environment, they agreed that there was never a break from worrying about food and body. One participant reflected: ‘When I started being competitive…that’s when I realized that it was important… it’s not just two nights a week, it’s almost every night, where you have to look good and eat healthy’ (P1, 17). This constant pressure created a relationship with the body that most participants believed to be negative.

Although all participants claimed to love the art of dance, many participants did not love the competitive environment. One participant commented: ‘The actual competitive atmosphere was never something that was very encouraging, it always kind of brought me down more than it made me feel good about myself’ (P12, 18). The competitive environment created numerous insecurities and pressures that dancers did not experience in recreational dance. Some participants believed that the added stress and overwhelming negativity of the competitive environment diminished their love for dance.

#### Ideal dancer’s body

Most dancers believed that in competitive dance the ideal dancer’s body is customary, important, and useful. The ideal dancer’s body was described as:

The tight tight tummy, the abdomen lines, the really really small chest, no curves, no bumps on your waistline, the thin tight compact thighs, your arms don’t jiggle, when you wear a top you don’t have rolls coming off of your straps at the back…everything is just basically tight and compact and stuck together.

(P2, 15)

Some participants believed that the ideal dancer’s body was starting to move away from being thin and towards being fit. This switch, however, did not produce a different relationship with how dancers felt about their bodies. Using the word ‘fit’ instead of ‘thin’ did not produce less pressure or expectation on dancer’s bodies. Striving to be fit created increased pressure for dancers to have minimal body fat and increased muscle definition. Participants also questioned if they should be aiming to achieve fitness, thinness, or both.

Although an ideal body image existed for all forms of dance, participants believed there was a distinguishable ideal for ballet. Participants believed that ‘ballerinas had the most stereotype placed on their bodies, whereas other dance styles were not as strict’ (P6, 17). Dancers understood that if you did not have the ideal ballerina body, ‘tall, skinny, long neck, the tiny little torso, the busty back and arched feet’ (P12, 18), you moved on to another style of dance; participants did not resist this notion. One participant commented: ‘People that are bigger or a bit more muscular will move on to hip hop or contemporary’ (P1, 17). It was generally believed that the ideal dancer’s body was more realistic to obtain if you were not a ballerina.

Regardless of the ideal that dancers were striving to obtain, or maintain, the amount of stress and pressure felt by participants was consistently high and shaped negative body image discourse. Dancers believed that this negative body image discourse would follow them forever. One participant commented: ‘It has now become my permanent body image’ (P1, 17). Participants believed their negative body image was something they would always internalize, and the ideal dancer’s body was a body they would always strive for.

#### Mirrors

Mirrors are a powerful teaching tool in the world of dance that draw an extraordinary amount of attention to the body. Dancers train in front of mirrors for hours each day to observe and evaluate their technique and skill. Participants consistently explained how they would look in the mirror and scrutinize every inch of their bodies. This constant scrutinizing and examining would generate negative thoughts and produce hate. One participant commented:

You just start to pick on little things and you just start to really dislike those little things…we’re constantly in front of a mirror, for hours in a day…so you’re always looking at those little things…just putting hate in the back of your mind.

(P9, 16)

The negative thoughts and self-talk participants engaged in was consistent and similar. Looking in the mirror initiated thoughts like, ‘I need to eat less, this needs to be flatter and I hate that, hate that, hate that’ (P5, 18). Participants also recognized that these negative thoughts transferred into their meals: ‘If I looked in the mirror and thought negative thoughts then I would keep those thoughts going into my meals’ (P1, 17). Mirrors optimized the critique of the body and participants were unable to escape the image being reflected.

#### Dance attire and costumes

Dance attire and costumes are another large part of a dancer’s life that shape their relationship with body and perpetuate the ideal dancer’s body discourse. Dance attire is generally tight, revealing, and bare much of the body; it is created for the ideal dancer’s body. When dancers cannot attain the ideal, and are not comfortable exposing their bodies, negative body image is often produced.

Participants believed ‘if you want to be a good dancer you have to be able to show everything off’ (P2, 15). Participants believed it to be a requirement for the body to be exposed both in class and at competition to be viewed, corrected, and judged. This made dance the primary environment where participants felt pressure to look good. One participant explained: ‘In my leotard and tights everything is out there, and it’s exposed…I guess it’s more the studio that I feel pressure to look good’ (P1, 17). Participants often spoke to how vulnerable they felt in their dance attire because every crevice of their body was visible.

Dance costumes and costume season also produced a sense of vulnerability and stress for participants because they worried about how their bodies would appear in their costumes. One participant commented: ‘When we do have to try on costumes there’s definitely a bit of nerves coming on to make sure it fits and it’s not too tight… costume season is nerve racking’ (P1, 17). Costume season is a time where the body is increasingly scrutinized by dancers as they pick their bodies apart in every single costume. If dancers believed the costume was not flattering on them, it resulted in stress and negative thoughts about their bodies. This happened frequently because costumes were often not custom made to fit each individual body, but instead costume designers and coaches attempted to fit a variety of body shapes and sizes into a few model prototypes. One participant explained:

There are 4 sizes for 4000 different body types, so some costumes could look beautiful on some dancers and then other costumes would look bad on me …costumes made you really pick yourself apart … really different places like your armpits or your legs… different things that you wouldn’t look at, costumes really accentuated, and sometimes you would get really self-conscious.

(P12, 18)

Dancers noted how costumes were supposed to fit unanimously, and it was stressful when the costume was less flattering on their body when compared to another dancers.

When dancers did not like how they looked in a costume, they were motivated to change their body before competition and justified the means of doing so. One participant shared: ‘I would tell myself it’s fine I can skip this meal and fit into my costume and look better on stage’ (P8, 18). Participants also stated that they felt better on stage when costumes covered more of their bodies.

### Parents

The influence of parents on the dancer’s relationship with body was seen by participants as being less significant than that of their environment, peers, or coaches. The influence of parents often went unrecognized by participants, with many commenting that their parents did not impact their relationship with body at all. It became apparent, however, that parents did create dominant and competing discourses about body image that influenced the relationship dancers had with their bodies. Through body monitoring, body talk, and the discursive struggles that parents create, this contradiction was illustrated.

#### Body monitoring

Although most participants disclosed that their parents did not comment on their bodies directly, many revealed how their parents, and other parents, discussed the bodies of other dancers in their presence. For example, parents would comment negatively on dancers getting ‘bigger’ over the summer or looking too thin. Participants gave meaning to the comments their parents made regarding the bodies of other dancers and translated them into messages about what the ideal dancer’s body should look like. Irrespective of discussion surrounding weight loss or weight gain, participants were equally impacted by this talk. Such talk affected participants negatively because it increased their awareness of the body and made them question if they were straying from the ideal. Parents may believe that it is not harmful when they discuss the body of other dancers. Dancers, however, are in a constant state of anxiety about their appearance and such comments easily inscribe and intensify themselves on their own body.

Participants further discussed how they are aware that parents talk about and observe the bodies of dancers, and how this creates insecurities for dancers when parents are present and/or observing them during dance class. Parents would often monitor rehearsals and practices closely from a viewing window, and this added additional elements of stress, insecurity, and surveillance.

Surveillance was always felt by participants, even when they were likely not being observed. This surveillance continued to be felt beyond the walls of dance:

I just think it’s important to work hard in every class and at competition because you never know whose watching…even on the streets…it’s not like people are going to dance on the street but say you have your hair in a bun and you’re walking to dance class, and somebody comes up to you and says ‘hey, you’re a dancer’.

(P7, 14)

Most dancers felt that they were always on public display and permanently visible to be observed and evaluated. Both the discussion and surveillance of parents contributed to self-monitoring of the body and resulted in practices that discursively shaped an ideal body image for young dancers.

#### Joking

Participants believed parents never talked about body directly, but when prompted further would go on to say that if parents did talk about the body, then it was only a joke. Jokes were perceived by participants as being acceptable and not ill-intentioned:

I don’t know if it is in a negative way but she (mother) will make like a joking comment like if you eat one more fry your body suit isn’t going to fit you…you won’t be able to get off the floor when you jump.

(P4, 17)

One of the participants acknowledged that joking is not a harmless form of delivery; the comments still held meaning and could be hurtful. This tool of communication is often an expression of criticism and shapes body image discourse that creates and enforcers the ideal dancer’s body.

#### Parents and support

Most participants recognized and appreciated when their parents were supportive, and often made a point of commenting that other parents were more critical than their own. Participants with supportive parents tended to report home as their safe space where they felt better about their bodies. One participant commented:

Like I mean if I am home …I am not worried about if I wear a sports bra or booty shorts or I am not wearing it for anybody to see what I look like…my mom’s not going to be like look you have a roll on your belly …so I feel like my parents are kind of that place.

(P2, 15)

Other participants commented on being comfortable at home; one participant described it as ‘secure’ (P3, 16), particularly in relation to her clothing. Participants with supportive parents also valued being able to turn to their parents for support and someone to talk to, claiming that this often kept them from engaging in eating disorder behaviours: ‘It is something that will be emotional for me as a dancer and as a person, but I can always look to my friends and parents for support’ (P1, 18). When the home environment was supportive, it provided a safe space that empowered dancers to resist disordered eating behaviours, even when faced with the strong forces at play in the world of dance.

## Coaches

According to participants, the influence of coaches on their relationship with body image was one of the most significant. All participants commented that they spent several hours a day with their coaches and viewed them as leading authority figures and role models in their lives. Participants largely complied with the commands of their coaches and were especially eager to please them. All participants also believed that coaches had a great influence over how they felt about their bodies:

Your coaches are the people you look up to…so they’re the people that tell you whether you’re doing good or whether you look bad and they’re the ones who basically have the final say on who you are as a dancer…as soon as someone you look up to, who is supposed to mentor you and coach you, kind of turns you down… that’s when your opinion of yourself completely changes.

(P12, 18)

Coaches were a large part of participants’ lives and highly valued and trusted by participants. It was often difficult for participants to recognize when the ways of knowing being formed by coaches were harmful because of trust, respect, and/or fear. This also made it difficult for participants to resist adopting the practices and beliefs of their coaches. The ways coaches discursively produced the ideal body manifested itself in several ways.

### Choosing how the body is displayed

Since correcting the body is a large part of dance, tight clothes are necessary to reveal the body. One participant commented: ‘You can’t wear baggy shirts and stuff … we are so high up in competitive that they (coaches) want us to be wearing all tight clothes so they can see every part of our bodies to correct it’ (P4, 17). Several studios even enforced dress code policies to deter baggy clothing, which created tensions for many dancers. One participant described the dress code at her studio, which only allowed dancers to wear a bra top and ‘bootie shorts’ or a body suit with no tights. She explained that because of the dress code her studio lost a lot of dancers: ‘So that year…I would say 6 people from my group quit and 6 people from the older group quit…and then we lost our whole adult group…because nobody wants to feel uncomfortable at dance’ (P2, 15).

This same issue of choice presented itself with costumes. Participants felt more confident about how their bodies were displayed on stage when they had a say in costume design. Most participants, however, expressed frustration with the lack of negotiation of power between coaches and dancers during this process. Comments arose like: ‘Group dances were entirely picked by the choreographer…so if you did not look good in the costume that was not something you could change unfortunately’ (P12, 18). It was often stated by participants that this was the way things were and there was no point in speaking up about it because ‘she is the teacher so if she says this is what you’re wearing it’s what we’re wearing’ (P8, 18). Communication was generally hierarchical and one-way. Some coaches even created rules against speaking up. One participant explained: ‘My dance teacher created a rule that if you didn’t like the costume the costume was taken away and you weren’t in the dance anymore’ (P12, 18). Such rules and practices created the belief for dancers that they are unable to comment on matters that affect their own bodies.

### Shaping the body

Most participants believed that their coaches were positive role models regarding shape, weight, and body. When participants were discussing scenarios that could portray their coach negatively, they would often attempt to defend their coach:

One time…my teacher wasn’t supposed to…she wasn’t trying to be negative but she just made a comment that my one friend had more of like a man body…she was more tall and wide and strong then you know petite and feminine and that really negatively affected her.

(P5, 18)

As interviews progressed it became evident that despite the participants’ beliefs, the body discourse perpetuated by coaches was predominantly negative.

Negative body related comments made by coaches were not uncommon. For example, one participant explained:

She (ballet teacher) made a comment about her weight in front of the whole class saying about her stomach being like ‘oh if you can’t lose it you got to suck it in’…obviously that had a very big impact on her and she was really upset by that for a long time.

(P6, 17)

Another participant described a comment by her ballet teacher:

Our ballet teacher said we had large thighs one time and that impacted a couple of my friends pretty negatively… like she wasn’t calling us fat or anything but…nobody really liked it that much…I don’t want big thighs…that’s not good…ballerinas aren’t supposed to have big thighs.

(P5, 18)

Weight and body based remarks communicated to all dancers that the thin body is valued, and excess weight impacted the ability to dance competitively. Comments from coaches often disciplined the fat body and resulted in dancers working to construct a thinner body that was closer to the ideal.

### Validating the thin body

Coaches validated the thin body in several ways. One of the ways was choreographing dances around specific dancers, or what some participants deemed ‘the favourites’. One participant commented:

They (the thin girls) are front and center…the bigger girls in the back… half of them are better and half of them I would say are equal to some of the self-conscious girls but… because they don’t look like what they want them to, the teacher would favour the girls that look better.

(P2, 15)

This practice led dancers to believe that the girls being chosen by coaches to be ‘front and center’ had the body type that was desirable enough to be seen.

Coaches further validated the thin body through compliments that rewarded participants for their appearance. Participants often expressed those compliments on their bodies from coaches made them feel good about themselves: ‘Of course I say complimented…like oh you have a great stomach let’s show that off…I’m going to put you in a half top for this dance’ (P5, 18). Another participant similarly described: ‘One time I was going across the floor…she said that my legs looked toned today…that makes me feel good’ (P11, 14). A coach complimenting the body based on appearance that fits the ideal reinforces the stereotypical dancer’s body. It sends the message to dancers that having a body thin or toned enough to show off is valuable and praised.

In general, the coach was the only instance where a positive comment on the body was valued by participants. When parents or peers would complement the body, participants would comment that it was meaningless and did not change how they felt about themselves. One participant commented: ‘If coaches could do something to build us up and to get us to stop thinking bad things about ourselves, I think that would help for sure’ (P9, 16). Coaches are in a position where they can make dancers feel good about their bodies and positively influence how their relationship with body and body image is shaped.

## Peers

Many participants remarked that the most enjoyable part of dance was the friendshipsthey had formed with other dancers. All participants spent most of their spare time at dance, and they were grateful to have friends at dance who could relate to their experiences. Participants did not tend to have close relationships with their peers outside of dance but consistently stated that they were very close to their dance friends. Such close relationships caused the influence of peers to be significant in shaping one’s relationship with body and body image.

### Peers and body

Participants talked about their bodies with peers more often than anyone else. Constant negative body talk was accepted as being normal by peers because everyone engaged in it: ‘Everyone, like everyone at my studio had negative things to say that they didn’t like about their body…no one had the perfect body’ (P5, 18). This negative body talk was habitual for participants and perpetuated a dominant body image discourse that produced self-consciousness and anxiousness about the body.

There was consensus among participants that dancers tended to talk positively about the bodies of others and negatively about their own bodies. For example:

If somebody were to take a group picture and somebody was like ‘omg everybody look at this girls abs’…we would all zoom in and be like omg you are so ripped, omg…so we are positive towards them…but if we were to say ‘ughhh I hate our top it gives us muffin top’… nobody would say anything.

(P2, 15)

### Peer comparison

Body talk, which is a dominant practice in the world of dance, often led to and/or was centralized around peer comparison about body shape, weight, and size. Body talk was primarily negative and led dancers to think and talk negatively about their own bodies: ‘I’m not like her, I’m not as good, I’m still too fat, I’m not as skinny as her (P8, 18), look at my thighs, they are bigger than yours…why do I have this roll and you don’t’ (P2, 15). Participants recognized that peer comparison had a large influence on how they felt about their bodies. They also recognized that it was a complex concept: ‘That’s the tricky part …you are always going to compare yourself to someone else no matter how much people compare themselves to you and no matter how much people envy the way you look’ (P8, 18). Although all participants engaged in peer comparison, the participants who self-admittedly claimed to not be close to the ideal body image engaged in increased peer comparison. They were also more self-conscious about their bodies, which led to an increased desire to compare and change the body.

Competition season further increased the amount of peer comparison among participants as the body was being shaped and prepared to be presented on stage. From the time the measuring tapes came out, tensions seemed to rise among participants. One participant commented: ‘When costume time comes around the measuring tapes come out and I think a lot of insecurities also come out’ (P10, 14). She went on further to explain: ‘Especially when they say your numbers’ (P10, 14). Participants were often measured in front of their peers, and it was not uncommon for peers to record the measurements for the costume designers. This situation created stress for participants as they worried about their measurements and how they compared to the measurements of their friends. This caused dancers to judge and compare their bodies based on the number of inches attached to each part.

When at competition, peer comparison increased as the pool of dancers grew, and winning dancers enforced the endeavor to achieve the thin ideal:

If you were to go to a competition and you’d see the girls that ended up winning everything are the girls that can wear a crop top and like underwear and have the ab lines…and the girls that have a hip line and their legs are like spaghetti….so it is more like you are trying to look like the girls that are winning.

(P2, 15)

By observing the body types of winning dancers, participants connected being thin with being successful. This comparison and connection were reinforced by social media.

Social media now consumes the lives of many adolescents, and, as a result, peer comparison has extended beyond the walls of dance class. The reason dancers wanted to look like the dancers on social media was because these dancers were explained as having the ‘famed body type’ (P12, 18). This was the body type participants believed was needed to be a professional or successful dancer. One participant commented that seeing professional or successful dancers on social media generated comparison: ‘You start comparing yourself to people who are more successful than you and then you compare leg length and arm length and body size…so that kind of changed how I see myself’ (P12, 18). Most dancers competing at a high level are striving to dance professionally and turn to social media to see what this entails. For participants, social media displayed a profound relationship between thinness and success, which most dancers internalized.

### Peers and joking

Discussion around body between peers was often perceived as a joke. Similar to parents, participants perceived jokes as being acceptable and harmless. For example, one participant commented: ‘My peers at dance we kind of …joke around I guess about our bodies…if there was something about our bodies that we didn’t like about it we would kind of just make fun of it in a joking way’ (P5, 18). Comments about the body such as, ‘I have a food baby’ (P1, 18), ‘I feel bigger today’ (P1, 18), and ‘I wish I was that skinny’ (P8, 18), were all presented by participants as being jokes. These comments, however, hold meaning about what is valued and acceptable practice, reinforce the thin body ideal, and can negotiate beliefs about body and body image. Additionally, perceiving body talk to always be a joke conceals the importance and impact of such talk. This can be detrimental for the dancers who need help and are actually using joking to express early warning signs of EDBs.

### Peers and support

With all the cultural forces in the world of dance that pressure dancers to conform to ideals and often unobtainable expectations, dancers expressed the critical need for having support systems. When asked how participants managed to resist or recover from EDBs, participants often commented it was their friends. One participant commented:

We all have those days and we all feel crappy and feel unconfident about ourselves…there still is the negative that is there, and it is always sort of part of our dance experience to feel uneasy about ourselves, but then the great thing that we can do is help each other and support each other.

(P1, 18)

It is critical that dancers feel supported by their peers and that dance environments encourage the establishment of peer support. Strong relationships with peers at dance can help with resistance and recovery of EDBs.

## Discussion

It has long been known that obsession with thinness and body image is culturally produced [9, 10, 12, 15, 110]. In relation to this study, there was consensus among the dancers interviewed that they felt the pressures of having to conform to the sociocultural expectations present in the world of dance. Although the thin ideal, media/social media, and objectification are factors for the development of EDBs in all young girls, the findings from this research display that participation in the world of dance magnifies these elements [33, 66]. This finding is similar to that of the current literature, which emphasizes that adolescent girls who participate in aesthetic sport are at the highest risk for development of EDBs [111–116]. The literature proposes that this is because of the extreme closeness between aesthetic sport and the predominant ideal of beauty, as well as the link between peak performance and a thin body [117, 118]. These proposed reasons were supported by this research. When studying dancers specifically, the findings of this research align with the current literature in terms of objectification, competitiveness, and weight related pressures from coaches and peers in the world of dance [66–69, 77, 80]. Further, these contributors shape the dancers’ relationship with body and body image and can contribute to the development and maintenance of EDBs. Additionally, dancers’ relationship with body is impacted by the influence of parents, coaches, peers, and the competitive dance environment.

### Parents

It was found that through talk, body monitoring, joking, and support parents shape the dancer’s relationship with body and body image, as well as contribute to the creation of dominant and competing discourses about body image. This is similar to the findings in the literature related to parental influence on the body image of adolescent girls [56, 57]. In this study, participants with supportive parents reported home as their safe space where they felt better about their bodies. This is congruent with current literature, which found that adolescent girls who reported having high levels of family support were shown to have higher levels of body satisfaction and lower levels of disordered eating compared to girls with lower levels of perceived family support [56].

Body monitoring was predominantly how parents influenced how dancers felt about their bodies. Parents often talked about the bodies of other dancers in terms of appearance and thinness versus heaviness. This signaled to dancers that the body is constantly under surveillance and reinforced that there is an obvious ideal that the body is being compared against. Dancers were aware that parents talk about the bodies of dancers. This created insecurities for dancers when parents were present and/or observing them while in dance class. Parents often observed dance classes through a viewing window, which added additional elements of stress, insecurity, and surveillance. Perhaps an alternative to the viewing window or less frequent observation of parents would make dancers feel more comfortable. If parents were less critical of the body, however, this would likely create an environment where the observation of parents would not be as stressful for dancers.

Parents may believe that it is not harmful when they discuss the body of other dancers. Dancers, however, are in a constant state of anxiety about their appearance and such comments can easily inscribe and intensify themselves on the dancer’s body. Further, it is not a harmless form of delivery when parents talk about food or body in the form of a joke. This tool of communication is often an expression of criticism and shapes discourses related to body image. Jokes constitute knowledge for dancers about what is acceptable behaviour in terms of appearance and diet. Humour often passes unnoticed but is in fact discursively forming the dancer’s relationship with their body image. This was similar to the findings in the literature that parental weight-teasing influenced eating disorder development amongst adolescent girls [58, 59].

Parents need to be aware of how they respond to their daughter’s questions and comments about their body and how they can be more supportive. The often silent response from parents discursively shaped negative body image and disordered eating discourse in dancers. In general, parents could be more aware of how and why they talk about their children’s body or the bodies of other girls/dancers. Increasing awareness for parents on how they create and perpetuate body image discourse could be a constructive first step in changing the dominant discourse in dance culture.

### Coaches

The findings illustrated that coaches significantly impacted the participants relationship with body and body image as they are influential role models for young dancers. This finding aligns with Francisco et al. [2], who also found coaches to be the strongest influencers upon young dancers. Coaches primarily contributed to discourses related to body image by how they displayed, shaped, and validated the body. Given the challenges participants noted with wearing skin-tight clothing for rehearsal, studios could attempt to create a balance between clothing that shows dance movements and clothing that exposes every part of a young girls body. Studios should reconsider dress codes/clothing policies with this in mind. Taking away a dancer’s choice to wear clothing they are comfortable in contributes negatively to a dancer’s relationship with their body. This lack of choice created constant anxiety for dancers, which they were rarely able to escape. The participants clearly stated that they felt more comfortable, confident, and empowered to develop a positive relationship with their body when they were able to choose their clothing.

This same issue of choice presented itself with costume design. When dancers had a say in the costume design process, they felt more confident about their bodies. It is important for coaches to include dancers in the costume design process and be more considerate of the bodies of all the dancers who will be wearing the costume. It is a simple way for coaches to engage dancers in matters of their bodies, help dancers feel confident, and empower them to have an opinion on the way their body is displayed. It is also important that coaches promote and encourage dancers to speak up about how they feel about their bodies in costumes or otherwise. The findings illustrated that coaches often discouraged, prevented, or undermined dancers speaking about their bodies. This can result in dancers believing that their body is for the consumption of others and their voice is not valued. This also often silenced dancers.

As stated in the findings, coaches contributed to discourses that perpetuated negative body image through their attempts to create the ideal dancer’s body. Dancers received feedback from coaches and adjusted their bodies accordingly in hopes of receiving their validation. In this feedback, participants heard that coaches only validated the ideal/thin body. One recommendation to begin making a cultural shift is for coaches to increase the acceptance of different body types in the world of dance. Coaches could start to do this by validating and displaying dancers with diverse body types. This could result in dancers being more accepting of both their own bodies and the bodies of other dancers. Participants noted that a variety of shapes, sizes, and weights can be successful in dance, however, they do not receive this message. Participants believed showcasing successful dancers who do not meet the traditional ideal is one way to assist in tearing down this stereotype. If girls with a variety of body types are not welcome, and therefore not present in the world of dance, the stereotype will perpetuate. If coaches begin displaying dancers with a variety of body types at competition who produce success, this could interrupt the stereotype that only dancers who have the ideal dancer’s body can be successful. The dominant image of an ideal dancer’s body could begin to shift and valuing extreme thinness in the world of dance could be disrupted.

### Peers

Dancers helped each other make sense of their experiences in the world of dance through body talk, comparison, and support. Participants claimed that the beliefs, values, and practices of all dancers are mostly the same. This similarity in beliefs, values and practices is because it is among peers where dancers largely formed an understanding of their body and body image. Dancers talked about their bodies with their dance peers more than with parents, coaches, or peers from school. This aligns with the finding that among adolescent girls generally, peers influence social norms and create social standards for appearance and behaviour [60]. Dancers also expressed the critical need for having peer support. This is congruent with the general literature on adolescent girls and eating disorders, which found that perceived peer support contributes to having a positive body image and was a protective factor against disordered eating [60]. High levels of peer support among dancers are important in improving body image and in turn reducing the risk of EDBs [56].

It was found that dancers are trained to admire the bodies of their peers but not to admire their own. This resulted in peer comparison being a dominant practice in the world of dance. The findings displayed that dancers did not feel that they were in an environment that empowered them to talk or feel positively about their own bodies, and that negative body talk was accepted as the cultural norm in dance. If coaches, parents, and dancers tried to become aware of when negative talk is occurring and support each other in shifting this talk in a positive direction, dancers may begin to think and talk more positively about their bodies. If dance culture shifted to create space for positive body talk and thoughts, it is possible that dancers would see that all bodies have value. It could also result in dancers being less vulnerable to the images presented to them of other dancers both in person and in the media. If dance culture continues to promote the dominantly negative thoughts and talk about the body, discourses that contribute to a negative body image will further perpetuate a problematic dance culture.

### Environment

Environmental elements in the world of competitive dance include mirrors, dance attire, costumes, and the image associated with the ideal dancer’s body. These elements shape the dominant ideal dancer’s body and body image related discourse that contribute to the beliefs, values, and practices of dancers. Although dancers cannot avoid their environment, the way they interact with these environmental elements does not need to be unhealthy or negatively shaped. It is likely that if a healthier culture of dance was created, the environmental influencers present in the world of dance would be less impactful.

Mirrors, for example, are a main teaching tool in the world of dance that were found to produce negative body image. Dancers observed, examined, compared, and scrutinized their bodies in the mirror for hours each day. These behaviours resulted in negative thoughts that, in turn, generated self-hate among participants. Even when dancers were not in front of the mirror, they continued to feel its reflection and surveillance weighing on them. Participants believed that mirrors were both inescapable and detrimental, but not because they were the root of the problem. The root of the problem was their negative body image and the overall negative relationship they had formed with their bodies. According to the findings, competitive dance culture generally creates dancers that identify with unhealthy beliefs, values, and practices in relation to body and body image, which intensifies when the mirrors are present. It is the sociocultural values in dance that promote the negative thoughts and disembodiment that dominate when dancers stand in front of the mirror. If dancers were in a dance culture that produced positive body image, the presence of mirrors would likely not be as daunting or produce as much negativity and hate. Similarly, dance attire and costumes are an environmental factor that perpetuate the ideal dancer’s body discourse. Dance attire and costumes are generally tight, revealing, and bare much of the body. They are created for the ideal dancer’s body. When dancers cannot attain this ideal, and are not comfortable exposing their bodies, negative body image is often produced.

Costume season created additional stress and anxiety for dancers because they worried about how their bodies would appear in their costumes. Costumes are often not custom made, but instead costume designers and coaches attempted to fit a variety of body shapes and sizes into a few model prototypes. Dancers noted how costumes were supposed to fit unanimously, and it was stressful when the costume was less flattering on their body when compared to another dancer. It may be beneficial for studios to consider having their costumes custom made to fiteach individual body. This would eliminate the stress dancers feel to fit into a mold that conflicts with their body shape and size. It would also eliminate the inconsistency of how the costume appears on each dancer, which often resulted in dancers feeling pressure to change their body. Based on the findings, dancers strongly believed that not only should consideration be given to the bodies of each dancer when designing costumes, but that coaches should also consider theamount of bare skin the costume exposes. Some dancers spoke to costumes that allowed for proper adjudication of the body without exposing a large amount of bare skin. Costumes that are extremely revealing also tend to be objectifying and sexualized. This can have long term implications for how dancers experience their bodies. Dancers learn from a young age that the body is meant to be exposed for the consumption of others and adjudicated based on thinness and attractiveness.

Dancers also spoke to being measured for costumes in the presence of others, which created anxiety about the measurements that would be attached to them. Participants expressed the extraordinary amount of insecurity and anxiety generated when they were measured in groups and when their peers knew their measurements. Measuring dancers in private and keeping their measurements confidential could help offset this anxiety. If the culture of dance, however, placed less emphasis on achieving the ideal dancer’s body, the insecurity and anxiety dancers felt while getting measured may diminish. In general, the dance environment creates many barriers that make it difficult for dancers to confidently shape a positive relationship with body and body image. These environmental elements, however, are predominantly negative because of the culture in which they are situated.

## Conclusion

The use of feminist poststructural discourse analysis provided a critical approach to explore the beliefs, values and practices related to the body and body image of young girls in competitive dance. Through interviewing twelve young dancers, it was found that all participants experienced negative physical, mental, and/or emotional repercussions throughout their competitive dance experience. It was also determined that environment, parents, coaches, and peers largely shaped the dancer’s relationship with body and body image in the world of dance. These influences generated and perpetuated the dominant negative body image discourse that dancers were often unable to resist, and consequently their relationship with body and body image suffered.

The findings of this research contribute to the current gap in the literature, which lacks a qualitative and critical approach to understanding the intricacies of EDBs in young girls who are also competitive dancers. Although the thin ideal, media/social media, and objectification are factors for the development of EDBs in all young girls [33, 66], the findings display that participation in the world of competitive dance intensifies these elements. The findings also provide a first-person account of young girls’ experiences in the world of dance that is lacking in current literature.

Implementing prevention efforts targeted at strengthening the relationship with the body is critical to reducing EDBs in adolescent girls who participate in competitive dance. For substantive change to occur, however, the world of dance needs to focus on generating a cultural shift that challenges the status quo and interrupts the beliefs, values, and practices that keep EDBs thriving in competitive dance culture. Although changing the culture of dance is a multi-level phenomenon, and there are many challenges to creating such a shift, disrupting the problematic everyday practices of dancers has the potential to change the dominant body and body image discourse they currently experience. Such change could significantly improve the health outcomes for young girls in the world of competitive dance.

## Supporting information

S1 Appendix (DOCX)
