# Supplementary material for: Dancing in a culture of disordered eating: A feminist poststructural analysis of body and body image among young girls in the world of dance
Source: PLoS One. 2022 Jan 12;17(1):e0247651. doi: 10.1371/journal.pone.0247651 (PMC8754298; doi:10.1371/journal.pone.0247651)
Supplement: S1 Appendix — (DOCX) [file pone.0247651.s001.docx]

Appendix A: Semi-Structured Interview Guide

Hello,

I am Nicole Doria, the primary researcher for this project. I am a former competitive dancer myself, and have lots of experience talking about dance, food and the body. I am interested in hearing about your experiences in the world of dance and how these experiences have shaped your relationship with food and your body. I have questions prepared, but please think of this interview more as friendly conversation.

To clarify, for the purposes of my research, the world of dance has been defined as a competitive culture with its own language, thoughts, communications, actions, beliefs and values. It is inclusive of dancers, dance teachers, the dance environment (mirrors, ballet bars, music), dance attire, costumes, stage makeup, peers, parents, judges/adjudicators, and the images associated with the ideal dancer.

Do you have any questions before we begin?

**Demographic Information:**

Age:

Training location/Studio:

Years of training as a competitive dancer:

**Questions:**

1. Can you tell me about your experience in the world of dance?

Potential prompts:

- How long have you been dancing?
- What types of dance do you do?
- Where do you dance?
- Were you always in competitive dance?
- Have you danced at other studios?

*How did that make you feel? How did you feel about that situation?

1. Can you tell me how the world of dance has shaped how you talk about your body?

Potential prompts:

- How do you talk about your body at dance? How do others (parents, peers, coaches)?
  - What influences this (parents, peers, coaches, mirrors)?
- How do you talk about your body in dancewear/dance costumes? How do others (parents, peers, coaches)?
  - What influences this conversation (parents, peers, coaches, mirrors)?
- How is this different then how you talk about your body outside of dance/in regular clothing?
- Do you ever talk positively about your body – can you explain?
- Do you ever talk negatively about your body – can you explain?
- Is how you talk about your body the same as how you feel about your body – can you explain?
- What is your reaction when you hear other people talking negatively/positively about your/their bodies?

1. Can you tell me about the ways the world of dance has shaped how you feel about your body?

Potential prompts:

- Tell me about the dance attire you practice and compete it makes you feel about your body?
- How is this different then how you feel about your body in regular clothing?
- How do the mirrors influence how you feel about your body?
- Who/what most in the world of dance influences how you feel about your body (peers/parents/coaches/dance wear/costumes/mirrors)?
- Are there places where you feel better/worse about your body? What are they?
- Have there been times in your life where you feel better/worse about your body? When/Where?
- Does how you feel about your body impact how you treat your body (dieting, unhealthy eating behaviours, etc.)? Can you explain

1. What things are important to you in terms of how your body looks and performs as a dancer?

Potential prompts:

- Do you think of your body as being whole or do you think of it as individual parts - how?
- How do you feel others expect your body to look and perform (coaches, peers, parents)?
- Do you feel more comfortable with your body at dance than you do outside of dance or vice versa?
- How has the world of dance influenced what is important to you in terms of how your body looks and performs?

1. Can you tell me how the world of dance has shaped how you talk about eating/food?

Potential prompts:

- How do you talk about eating/food at dance? How do others (parents, peers, coaches)?
  - What influences this conversation (parents, peers, coaches)?
- Is this different then how you talk about food/eating outside of dance? How?
- Do you ever talk positively about food/eating – can you explain?
- Do you ever talk negatively about food/eating – can you explain?
- Is how you talk about food/eating reflected in what you eat/your food choices? How?
- What is your reaction when you hear other people talking negatively/positively about food/eating?

1. Can you tell me about the ways the world of dance has shaped how you feel about eating/food?

Potential prompts:

- Tell me what you think about eating/food? How does this impact your food choices?
- Do you often eat the same foods – what are they?
- Do you think that you have different eating behaviours compared to your peers that aren’t in dance? What about your peers that are in dance?
- Do you think of eating/food differently at dance and at home?
- Do how you feel about eating/food impact what you eat?

1. Is there anything else about your experiences in the world of dance you would like to tell me about?
2. Do you have any questions before we finish?

*Thank you for sharing – I really appreciate it!*
